# Survival analysis in breast cancer: evaluating ensemble learning techniques for prediction

**DOI:** 10.7717/peerj-cs.2147

**Published:** 2024-07-10

**Authors:** Gonca Buyrukoğlu

**Affiliations:** Department of Statistics/ Faculty of Science, Çankırı Karatekin University, Çankırı, Turkey

**Keywords:** Conditional inference forest, C-index, Integrated Brier score, Prediction error curve, Random survival forest, METABRIC, German Breast Cancer Study Group 2

## Abstract

Breast cancer is most commonly faced with form of cancer amongst women worldwide. In spite of the fact that the breast cancer research and awareness have gained considerable momentum, there is still no one treatment due to disease heterogeneity. Survival data may be of specific interest in breast cancer studies to understand its dynamic and complex trajectories. This study copes with the most important covariates affecting the disease progression. The study utilizes the German Breast Cancer Study Group 2 (GBSG2) and the Molecular Taxonomy of Breast Cancer International Consortium dataset (METABRIC) datasets. In both datasets, interests lie in relapse of the disease and the time when the relapse happens. The three models, namely the Cox proportional hazards (PH) model, random survival forest (RSF) and conditional inference forest (Cforest) were employed to analyse the breast cancer datasets. The goal of this study is to apply these methods in prediction of breast cancer progression and compare their performances based on two different estimation methods: the bootstrap estimation and the bootstrap .632 estimation. The model performance was evaluated in concordance index (C-index) and prediction error curves (pec) for discrimination. The Cox PH model has a lower C-index and bigger prediction error compared to the RSF and the Cforest approach for both datasets. The analysis results of GBSG2 and METABRIC datasets reveal that the RSF and the Cforest algorithms provide non-parametric alternatives to Cox PH model for estimation of the survival probability of breast cancer patients.

## Introduction

Breast cancer is fatal disease and the rate of the disease is increasing day by day (Bray et al., 2018). Although there has been an enormous amount of studies about breast cancer and much made progress, it is still one of the leading causes of death amongst women. Yet, around 40% of cases can be preventable according to breast cancer statistics report (World Cancer Research Fund/American Institute for Cancer Research, 2018). The report also presents that there were 2.1 million estimated breast cancer cases worldwide in 2018. The World Health Organization (WHO) states that breast cancer is the most widespread occurring cancer globally as of 2021, accounting for 12% of all new cases annually in the world (World Health Organization, 2022a). The principal goal of the WHO Global Breast Cancer Initiative (2021) is the reduction of global breast cancer mortality by 2.5% per year to save 2.5 million lives worldwide by 2040 (World Health Organization, 2022b). Comprehensive breast cancer management, health promotion for early detection, and timely diagnosis are the three pillars to achieve this goal. This research will shed light on supporting the last two building blocks of the goal with the corresponding analysis.

Survival rate calculation is important in order to measure the impact of treatments and the survival of a patient is affected from various inputs. Tumor conditions, patients’ situations, treatment or every other covariate may have an impact of the survival rate. Therefore, modelling survival outcomes with the various factors can aid clinicians to have better diagnosis. Estrogen receptor status, progesterone receptor status, age and tumor stage are another important predictive prognostic features (Siddarth et al., 2016; Singh et al., 2014). The former two steroid hormones crucial in causing hormone related breast cancer time-to-event analysis also known as survival analysis is one of the most commonly utilised techniques in breast cancer research.

Time-to-event analysis is a well-known statistical method for analysing time-to-event data. The terms of survival time and time-to-event are utilised synonymously throughout this study. The main interest lies in the time, it takes from a given baseline for an event of interest to happen, and the variables related to the event of interest. The time-to-contracting cancer, recurrent of a disease or, death can be some examples of the time-to-the-event. The most traditional method of analysing this type of data is the Cox proportional hazards (PH) model.

The method presented by Cox (1972) is a semi-parametric method with the incorporating of independent features, and this method is known as the Cox proportional hazards model. The Cox PH model is well-designed for small datasets and may not be the optimal way of analysing medium or high dimensional data settings. Alternatively, machine learning techniques that intrinsically deal with complex data structure regardless of data size.

Machine learning models have been proposed to make prediction for different purposes in variety of fields such as health (Buyrukoğlu, 2021), cybersecurity (Savaş & Savaş, 2022), agriculture (Buyrukoğlu, Buyrukoğlu & Topalcengiz, 2021), sport science, *etc*. (Richter, O’Reilly & Delahunt, 2021). Machine learning approaches accommodating with the high-dimensional structure, correlated features, nonlinearity in the data, have been adapted to cope with event times data (Wang, Li & Reddy, 2019). One of the most popular machine learning models is random forest proposed by Breiman (2001). It is an ensemble tree-based learner. Random survival forest (RSF) approach, introduced by Ishwaran et al. (2008), is an extension of the random forest and an ensemble tree method for analysis of right-censored time-to-event data. Another bagging ensemble algorithm is the conditional inference forest (Cforest) proposed by Hothorn et al. (2004). This method is suggested to aggregate survival trees to improve predictive capability of model. The last two methods are also called ensemble learning methods. RSF is the second commonly employed algorithm after the traditional method (the Cox PH model) in survival data problems. RSF is an ensemble tree-based learner which is specifically designed to analyse time-to-event/survival data, and Cforest is a significant decision tree class based on statistical tests which is improved for event time trees as a special case. Although RSF can be considered as an alternative to Cox PH model, it can have bias towards inclusion of variables with many split points and therefore Cforest methodology can handle this selection bias *via* two-step split procedure with the implementation of hypothesis tests (Strobl et al., 2007; Wright, Dankowski & Ziegler, 2017; Das, Abdel-Aty & Pande, 2009). Cforest algorithm is less known and less utilised algorithm compared to RSF and other machine learning algorithms. This study will focus on the most popular traditional method (Cox PH model), the second commonly used algorithm (RSF) and less popular method (Cforest) to analyse event time data in order to indicate their performances and superiorities. As the Cox PH model is well-known amongst clinicians, this may have low model performance. Alternatively, ensemble learning techniques can be replaced by this traditional technique, as they are available to employ in many software programs, easy to understand and model fit.

So far, some studies have compared the performance of the methods in the survival analysis framework in breast cancer studies. Abbass et al. (2011) specified the key factors affecting the prognosis of the breast cancer with RSF and Roder et al. (2012) investigated the age effects on survival for breast cancer patients. Nasejje et al. (2017) and Liu et al. (2021) compared the RSF and Cforest model performance with simulated event time datasets and predicted survival rate with real datasets. Although each model has its own superiority, the estimation methods can have impact on the model’s performances. To the best of authors’ knowledge, no previous study has distinguished between the bootstrap and the bootstrap .632 for estimation of the risk prediction performances of the models. The main contribution of this study is to demonstrate that ensemble learning algorithms do indeed have state-of-the-art predictive ability in practice on real breast cancer datasets and evaluate how their performances changes based on different bootstrap estimation methods. To achieve this contribution the objectives are listed:

There are three objectives of this study, which are:

• to compare the performance ability of the Cox model, ensemble (Cforest and RSF) models that are appropriate for censored and clinical data by predicting time-to-relapse of breast cancer shed light on how their performances differ based on estimating the prediction performance.
• to provide insight into the reasons of performance of the models (informativeness of the variables) the way they did.
• to illustrate the results of the model fits for the hypothetical patients.

The rest of the article is organized as follows: ‘Related Works’ reviews the recent works related to our study. ‘Datasets’ introduces datasets utilised for the analysis. ‘Time-to-event Analysis’ describes the models and the notations. ‘Evaluation’ gives details about the evaluation metrics utilised in this study. ‘Results’ gives the results from the analysis. Finally, in ‘Discussion and Conclusion’, a brief summary is given and the results are discussed with the potential extensions.

## Related Works

Some studies have focused on the comparison of the model performances in terms of breast cancer survival. Kurt Omurlu, Ture & Tokatli (2009) compared the performances of Cox model and RSF method with Monte Carlo simulation and a real breast cancer dataset. The authors made comparison across various sample sizes and three different splitting rules. The evaluation was made through C-index. Their dataset included 279 breast cancer patients with 23 predictors. The study determined the major risk factor for event free survival. It concluded that both methods had almost similar performances whereas the performances of RSF had slightly better in terms of C-index.

Lee et al. (2018) proposed DeepHit method, using deep neural network to understand the distribution of the event times. The method had some advantages such as making no assumptions about the stochastic process, allows the correlation between predictor and risk and coping with the competing risk. The authors made comparison of the proposed algorithm with Cox PH, RSF, DeepSurv, Adaboost, LogitR and also analysed the METABRIC dataset and the method is evaluated with C-index. It concluded that the algorithm had better performance than the other state-of-the-art methods in order to handle survival datasets.

Katzman et al. (2018) introduced DeepSurv method, which is a Cox PH deep neural network, and can take into account the interactions between predictors and treatment effectiveness. The method is performed *via* simulation studies and real datasets employed and shown that the performance of new method is as well as the other state-of-the-art survival methods based on the C-index values. METABRIC dataset is one of the datasets employed in this study. In addition to this, this method has the capability of handling the complex relationship between predictors and event risk. The authors concluded that the modelling capabilities of the method is to enable medical doctors to utilise it as a tool in their exploration and prediction of patients’ characteristics of event risk.

Ganggayah et al. (2019) compared the predictive performances of machine learning methods and they analysed a large breast cancer dataset from the University Malaya Medical Centre, Kuala Lumpur, Malaysia, 8,066 patients and 23 predictors with diagnosis of breast cancer between 1993 and 2016. The authors built predictive models *via* decision tree, support vector machine, random forest, extreme boost, logistic regression and neural networks. They compared the results based on AUC and identified the most important factors. They concluded that random forest yielded the highest accuracy among those utilised.

Moncada-Torres et al. (2021) made a comparison between CoxPH model and several machine learning algorithms, such as RSF, survival support vector machines, and Extreme Gradient Boosting (XGB) using breast cancer data from Netherlands Cancer Registry. They evaluated the methods using C-index. The authors concluded that the predictive ability of the machine learning methods had better C-index values; however, the best performance belongs to XGB algorithm due to its ability to deal with nonlinearities and complexity.

Tong & Zhao (2022) proposed a nuclear-norm-based deep survival algorithm (NN-DeepSurv). The nuclear norm part imputes the missing predictors and unified with the deep survival algorithm for right censored survival data. The algorithm is tested with the simulation studies and compared with other algorithms such as CoxPH model, CoxPH model with Lasso regression, RSF, DeepSurv and XGboosts and implemented METABRIC data and two more datasets. Their performances were evaluated through the C-index and found that their method had promising results.

Evangeline, Kirubha & Precious (2023) compared predictive performances of the Cox PH and RSF and DeepHit models utilizing the METABRIC dataset. It identifies the significant predictors for the event failure. Both DeepHit and RSF models gave better C-index values than the CoxPH model. The authors founded that the most important covariate is the relapse free status of the patients. Nevertheless, this variable could be accounted as survival output and could be considered as competing risk for the survival analysis.

Most of the studies so far either utilized a small dataset or had short follow-up time and none of the above studies interested in bootstrap methods. Our study focuses on predictive survival capabilities of the models and identifies the key covariates which can be directly associated with event time output, utilizing the CoxPH model, RSF and Cforest based on two different bootstrapping techniques and evaluate them *via* integrated Brier score and C-index. GBSG2 and METABRIC datasets are analysed.

## Datasets

There are two datasets utilised in this study: The German Breast Cancer Study Group 2 and METABRIC datasets. They are well-known datasets amongst researchers and one can reach them straightforwardly. So that the readers can repeat the analysis without trouble. However; the most important reason of choosing these datasets are that the datasets can be said quite balanced. Their event rates are 43.6% and 62.3%, respectively. If the datasets were unbalanced the model performances would not be trustworthy.

### German breast cancer study group 2

The German Breast Cancer Study Group 2 (GBSG2) is a multi-center randomized trial for comparison of six *versus* three cycles of methotrexate, cyclophosphamide, and fluorouracil (CMF) starting perioperatively. This study aims at investigating the extra effect of tamoxifen as adjuvant treatment in node-positive breast cancer patients treated with mastectomy. 686 individuals with node-positive breast cancer were randomized from 41 institutions between 1984 and 1989 and to receive either 2 years of hormonal therapy with tamoxifen (TAM) or no hormonal therapy. The event for the study is the recurrence of breast cancer during follow-up time.

The outcomes of interest of this study are censoring indicator (0- censored, 1- event) and recurrence free survival time (in days). A total of 299 patients out of 686 have the event during the follow-up period. Therefore, the event rate is approximately 43.6%. The explanatory variables are given in Table 1: hormonal therapy- a factor at two levels, baseline age of the individuals, menopausal status—pre and post, tumor size (in mm), tumor grade—at three ordered levels I<II<III, number of positive nodes, progesterone and estrogen receptor (in fmol) (Sauerbrei et al., 2000).

**Table 1:** Baseline characteristics of GBSG2 participants.

**Variables**	**Breast cancer recurred (Failed), *n* = 299**	**Breast cancer did not recur (Censored), *n* = 387**	**Combined, *n* = 686**
Hormonal therapy -yes^*^	94(31.44%)^**^	152(39.28%)	246(35.86%)
Age	53(10.86)	53.09(9.52)	53.05(10.12)
Menopausal status-pre^*^	119(39.80%)	171(44.19%)	290(42.27%)
Tumor size (in mm)	31.46(15.75)	27.68(12.84)	29.33(14.30)
Tumor grade-II^*^	202(67.59%)	242(62.53%)	444(64.72%)
Tumor grade-III^*^	79(26.42%)	82(21.19%)	161(23.47%)
Number of positive nodes	6.51(6.13)	3.84(4.58)	5.01(5.48)
Progesterone receptor (in fmol)	70.52(122.21)	140.49(242.86)	109.99(202.33)
Estrogen receptor (in fmol)	85.54(152.68)	104.53(153.08)	96.25(153.08)

**Notes.**

* Data are categorical, the rest are continuous.

** Data are mean (SD) or *n*(%).

Table 1 indicates the baseline characteristics of the patients. The mean of tumor sizes and the mean of number of positive nodes of those who had the event is evidently bigger than those who did not have the event.

In order to illustrate a construction of the three hypothetical new individuals is made. These hypothetic patients have the same variables as in the GBSG2 study with different ages: Their ages are 24, 53 and 7. The details of these hypothetical patients can be found in Supplemental Information Files.

### METABRIC dataset

This study includes long terms clinical outcomes of breast cancer patients along with their inherited genetic variation variables. It includes a composed set of over 2,000 primary tumours (METABRIC, Molecular Taxonomy of Breast Cancer International Consortium) (Curtis, 2012; Pereira et al., 2016). The dataset is publicly available on https://www.cbioportal.org/datasets. This study aims at investigating the clinical heterogeneity underlying the disease taxonomy. It includes the gene expressions, survival information, copy number of variations and clinicopathological details. The study recorded patients’ status if the patients were deceased or alive and/or if any recurrence happened up to 20 years (Rueda et al., 2019).

The overall patient status (living/deceased) and relapse of the disease (recurred/not recurred) are pooled and defined as new event indicator in order to provide consistency between the two studies. The event rate of this study is 62.3%. Therefore, the outcomes of interest of METABRIC study are the event status and event time (in days). The explanatory variables and their baseline characteristics are given in Table 2. The patients having missing observations in any variable are not taken into account for the analysis. Out of 2,509 observations, approximately half of them were removed from the dataset due to incompleteness. The analyses were done using 1,269 observations. The dataset is split into two: training (70%) and testing (30%) datasets for the analysis.

**Table 2:** Baseline characteristics of METABRIC participants.

**Variables**	**Had the event (Failed), *n* = 479**	**Did not have the event (Censored), *n* = 790**	**Combined, *n* = 1, 269**
CellularityModerate^*^	191(39.9%)^**^	302(38.2%)	493(38.8%)
CellularityHigh^*^	233(48.6%)	402(50%)	635(50%)
ChemotherapyYES^*^	117(24.4%)	162(20.5%)	279(22%)
ER.StatusPositive^*^	363(75.8%)	616(78%)	978(77%)
Neoplasm.Histologic.Grade2^*^	192(40%)	305(38.6%)	497(39%)
Neoplasm.Histologic.Grade3^*^	240(50.1%)	431(54.5%)	671(52.8%)
HER2.StatusPositive^*^	49(10.2%)	104(13.2%)	153(12%)
Hormone.TherapyYES^*^	282(58.9%)	479(60.6%)	761(60%)
Inferred.Menopausal.StatePre^*^	143(30%)	150(19%)	293(23.1%)
Lymph.nodes.examined.positive	1.04(2.39)	2.41(4.49)	1.89(3.89)
Mutation.Count	4.88(3.09)	5.87(4.17)	5.5(3.83)
Nottingham.prognostic.index	3.91(0.98)	4.26(1.09)	4.13(1.06)
PR.StatusPositive^*^	255(53.2%)	407(51.5%)	662(52.2%)
Radio.TherapyYES^*^	342(71.4%)	507(64.2%)	849(67%)
Tumor.Size	22.8(11.8)	28.1(16.5)	26.12(15.14)
Tumor.Stage2^*^	256(53.4%)	480(60.8%)	736(58%)
Tumor.Stage3^*^	23(4.8%)	90(11.4%)	113(8.9%)
Age	56.4(11.3)	62.8(13.4)	60.4(13.0)

**Notes.**

* Data are categorical, the rest are continuous.

** Data are mean (SD) or *n*(%).

## Time-to-event Analysis

### Cox PH model

The Cox PH model is a semi-parametric model and can be written as:

(1) \begin{eqnarray*}h \left( t \right) ={h}_{0} \left( t \right) \exp \nolimits (X\beta )\end{eqnarray*}

where $h \left( t \right) \text{}$ isthe expected hazard at time t, ${h}_{0} \left( t \right) $ is the baseline hazard, ***X*** is design matrix and *β* is the vector of corresponding regression coefficients. Partial likelihood function derived by Cox (1972) for the *i*thsubject can be written as:

(2) \begin{eqnarray*}{L}_{i} \left( \beta \right) ={ \left[ \frac{\exp \nolimits (X\beta )}{\sum _{i\in Y({t}_{i})}\exp \nolimits (X\beta )} \right] }^{{\Delta }_{i}}\end{eqnarray*}

where *t*_*i*_ and Δ_*i*_ are the censoring or event time and the censoring indicator for the *ith* individual, respectively. *Y*(*t*_*i*_) is the set of individuals who are at risk at time *t*_*i*_ , that is *Y*(*t*_*i*_) = *j*:*t*_*i*_ ≤ *t*_*j*_, the order of the event times.

While this model left unspecified the baseline hazard function, it requires some restrictive assumptions: Random censoring, proportional hazard assumption and linear covariates (Murphy & Kleinbaum, 1997).

#### Random survival forests

RSF is based on the classical random forest approach, which is a nonparametric machine learning technique used for building prediction of the risk model in time-to-event analysis. Implementation of them follows the same principles. Splitting rule and bootstrap samples are two key parts of the RSF, and randomization is done by two steps. To grow the tree, in the first step, randomly drawn bootstrap samples of data is utilised. In the second step, nodes on randomly selected predictors are split to grow the survival tree. Then, log-rank splitting rule is used to measure the quality of a split. Finally, the calculation of the random forest output is done with the mean of the individual tree predictors (Ishwaran et al., 2008).

Let ${\mathfrak{T}}_{b} \left( x \right) $ be the terminal node of subjects in the *b*th bootstrap sample with *x*. Let *c*_*ib*_ denote the number of times *i*th individual happens in the *b*th bootstrap sample. If the *b*th bootstrap sample does not include the *i*th subject, then *c*_*ib*_ = 0. Following Mogensen, Ishwaran & Gerds (2012), the counting process notations Coolen et al. (1996) are introduced here.

\begin{eqnarray*}{\tilde {N}}_{i} \left( s \right) =\mathfrak{I}({\tilde {T}}_{i}\leq s,{\Delta }_{i}=1); {\tilde {Y}}_{i} \left( s \right) =\mathfrak{I}({\tilde {T}}_{i}> s) \end{eqnarray*}

we know

\begin{eqnarray*}{\tilde {N}}_{b}^{\ast } \left( s,\mathbf{x} \right) =\sum _{i=1}^{N}{c}_{ib}\mathfrak{I} \left( {X}_{i}\in {\mathfrak{T}}_{b} \left( x \right) \right) {\tilde {N}}_{i} \left( s \right) ; {\tilde {Y}}_{b}^{\ast } \left( s,\mathbf{x} \right) =\sum _{i=1}^{N}{c}_{ib}\mathfrak{I} \left( {X}_{i}\in {\mathfrak{T}}_{b} \left( x \right) \right) {\tilde {Y}}_{i} \left( s \right) \end{eqnarray*}

where ${\tilde {N}}_{b}^{\ast } \left( s,\mathbf{x} \right) $ is the events those who do not experience until time *s* and ${\tilde {Y}}_{b}^{\ast } \left( s,\mathbf{x} \right) $ is the number at risk at time *s*.

Ishwaran et al. (2008) constructed the ensemble through the aggregation of the tree-based Nelson-Aalen estimators. Particularly, the Nelson-Aalen conditional cumulative hazard rate estimator is given by

\begin{eqnarray*}{\hat {\mathrm{H}}}_{\mathrm{b}} \left( \mathrm{t}{|}\mathbf{x} \right) =\int \nolimits \nolimits _{0}^{\mathbf{t}} \frac{{\tilde {\mathrm{N}}}_{\mathrm{b}}^{\ast } \left( \mathrm{ds},\mathbf{x} \right) }{{\tilde {\mathrm{Y }}}_{\mathrm{b}}^{\ast } \left( \mathrm{s},\mathbf{x} \right) } . \end{eqnarray*}

The ensemble the survival function based on the Nelson-Aalen estimator from random survival forest can be calculated as follows:

\begin{eqnarray*}{\hat {\mathrm{S}}}^{\mathrm{rsf}} \left( \mathrm{t}{|}\mathbf{x} \right) =\exp \nolimits \left( - \frac{1}{\mathrm{B}} \sum _{\mathrm{b}=1}^{\mathrm{B}}{\hat {\mathrm{H}}}_{\mathrm{ b}} \left( \mathrm{t}{|}\mathbf{x} \right) \right) . \end{eqnarray*}

### Conditional inference forests

Hothorn et al. (2004) defined the aggregated Kaplan–Meier curve of a new observation by the Kaplan–Meier curve of all subjects identified by the leaves including new subject. The ensemble survival function is given by:

\begin{eqnarray*}{\hat {\mathrm{S}}}^{\text{cforest}} \left( \mathrm{t}{|}\mathbf{x} \right) =\prod _{s\leq t} \left( 1- \frac{\sum _{b=1}^{B}{\tilde {\mathrm{N}}}_{\mathrm{b}}^{\ast } \left( \mathrm{ds},\mathbf{x} \right) }{\sum _{b=1}^{B}{\tilde {\mathrm{Y }}}_{\mathrm{b}}^{\ast } \left( \mathrm{s},\mathbf{x} \right) } \right) . \end{eqnarray*}

This function is asymptotically equivalent to the following function in case of the continuous survival function:

\begin{eqnarray*}\exp \nolimits \left( -\int \nolimits \nolimits _{0}^{t} \frac{\sum _{b=1}^{B}{\tilde {\mathrm{N}}}_{\mathrm{b}}^{\ast } \left( \mathrm{ds},\mathbf{x} \right) }{\sum _{b=1}^{B}{\tilde {\mathrm{Y }}}_{\mathrm{b}}^{\ast } \left( \mathrm{s},\mathbf{x} \right) } \right) . \end{eqnarray*}

Cforest assigns more weight on terminal nodes in which there are a considerable number of individuals at risk:

\begin{eqnarray*} \frac{\sum _{b=1}^{B}{\tilde {\mathrm{N}}}_{\mathrm{b}}^{\ast } \left( \mathrm{ds},\mathbf{x} \right) }{\sum _{b=1}^{B}{\tilde {\mathrm{Y }}}_{\mathrm{b}}^{\ast } \left( \mathrm{s},\mathbf{x} \right) } = \frac{1}{B} \sum _{b=1}^{B} \left[ \frac{{\tilde {\mathrm{Y }}}_{\mathrm{b}}^{\ast } \left( \mathrm{s},\mathbf{x} \right) }{ \frac{1}{B} \sum _{b=1}^{B}{\tilde {\mathrm{Y }}}_{\mathrm{b}}^{\ast } \left( \mathrm{s},\mathbf{x} \right) } \right] \frac{{\tilde {\mathrm{N}}}_{\mathrm{b}}^{\ast } \left( \mathrm{ds},\mathbf{x} \right) }{{\tilde {\mathrm{Y }}}_{\mathrm{b}}^{\ast } \left( \mathrm{s},\mathbf{x} \right) } \end{eqnarray*}

Conversely, RSF utilizes weights on entire terminal nodes equally.

## Evaluation

Several measures are available for the assessment of the fitted model in time-to-event analysis. Attention will be C-index, and Brier Score.

### Concordance index

The Concordance index (C-index) measures discrimination capability of a model, the proportion of pairs where the patient with the lower event time has the lower probability of survival (Harrell et al., 1982). The higher C-index measure indicates better prediction performance. The value of 0.5 represents the average performance of a random model, whereas the value of 1 represents that a model is perfectly capable of separation of individuals with different outcomes (Harrell et al., 1982; Harrell, Lee & Mark, 1996).

### Prediction error curves

Prediction error curves (PEC) evaluate the risk prediction models’ performance event times analysis. The Brier score is a weighted average of the squared distances between the observed and the predicted survival probability of the model. The weights approximately correspond to the probabilities of being uncensored. The weights may be predicted based on covariates in the model. PEC can be obtained through the time-dependent expected Brier score, defined as:

\begin{eqnarray*}BS \left( t,\hat {S} \right) =E({Y}_{i} \left( t \right) -\hat {S}(t{|}{X}_{i}))^{2}. \end{eqnarray*}

where ${Y}_{i} \left( t \right) $ is the indicator for individual *i* ( ${Y}_{i} \left( t \right) =I({T}_{i}\geq t)$), $\hat {S}(t{|}{X}_{i})$ is the predicted survival probability at time *t* for individual *i* with covariates *X*_*i*_. The expectation is taken with respect to the data of individual *i* on the test set. Comparison and assessment of the predictive performance of the different modelling methods on the same set of data can be provided through cross-validation.

The integrated Brier score (IBS) is the cumulative Brier score within the range of [0, *τ*] and is defined as follows:

\begin{eqnarray*}IBS \left( \tau \right) = \frac{1}{\tau } \int \nolimits \nolimits _{0}^{\tau }BS \left( u,\hat {S} \right) dW(u) \end{eqnarray*}

where *W*(*u*) isa function of weighting the contribution of the Brier score at individual time point, and *τ* > 0 is a time before the last event time.

#### Cross-validation

In case of the availability of only one data set to build a model and estimate the model performance, there are some methods to deal with the overfitting problems (Gerds & Schumacher, 2007): the apparent, the bootstrap cross validation and the bootstrap .632 estimates. Firstly, the apparent estimate resubstitutes the data of the all individuals which were utilised to build the models. Secondly, the bootstrap cross-validation estimate method splits the data into two parts: training samples and test samples. The samples may be either the with or without replace from the corresponding data. After that, the models are trained with the training data and tested *via* the bootstrap testing data. In the final step, the bootstrap cross-validation estimate of the prediction error is found through the mean of the testing data. Third, a linear combination of the first and the second using constant weight .632 comprises the bootstrap .632 estimate.

## Results

The Cox PH model, the random survival forest and the conditional inference forest methods were implemented in the GBSG2 and METABRIC studies. The primary aim was to compare the performances of the traditional statistical method and ensemble learning technologies to be able to decide if more flexible method outperforms the classical statistical method, and provides insight in breast cancer survival.

A summary of performance results obtained from the Cox PH model regarding GBSG2 dataset is presented in Table 3. According to the Cox PH model and based on the *p*-values of each variable, the parameters belonging *menopausal status, tumor grade, number of positive nodes* and *progesterone receptor* are the statistically significant parameters and these factors have an effect of overall survival status of the patients. The table also includes information about the effect size of the variables through the hazard ratios (the exponential coefficients). These results are obtained with the implementation of the function of coxph() in survival package in R (Therneau & Grambsch, 2000; Therneau, 2012).

**Table 3:** Cox PH model parameter estimation results for GBSG2 dataset.

	$\hat {\beta }$	$\exp (\hat {\beta })$	$se(\hat {\beta })$	*z*	Pr(>|*z*|)
horTh-yes	−0.2332	0.792	0.1528	−1.5262	0.127
age	−0.0211	0.9792	0.0111	−1.9042	0.0569
Menostat-Pre	−0.4387	0.6449	0.2126	−2.0636	0.0391
tsize	0.0052	1.0052	0.0047	1.1107	0.2667
tgrade-II	0.5108	1.6667	0.2738	1.8655	0.0621
tgrade-III	0.6155	1.8506	0.2957	2.0813	0.0374
pnodes	0.0433	1.0442	0.0086	5.0163	0
progrec	−0.0021	0.9979	7e−04	−3.222	0.0013
estrec	3e−04	1.0003	5e−04	0.5705	0.5683
Concordance	0.682	(se = 0.018)			
Likelihood ratio test	61.93	on 9 df	*p* = <6e−10		
Wald test	66.38	on 9 df	*p* = <8e−11		
Score (logrank) test	68.26	on 9 df	*p* = <3e−11		

A summary of Cox PH model parameter estimation result for METABRIC data is indicated in Table 4. It can be seen from the table that the parameter estimates of *HER2 status, Hormone Therapy, Menauposal status, Lymph nodes, Radiotherapy, Tumor size* and *age* are statistically significant parameters. Positive parameter estimates show higher hazard rate. If the parameter estimate is smaller than zero, the subject has a low risk in terms of having event.

**Table 4:** Cox PH model parameter estimation results for METABRIC data.

	$\hat {\beta }$	$\exp (\hat {\beta })$	$se(\hat {\beta })$	*z*	Pr(>|*z*|)
CellularityModerate	−0.0695	0.9329	0.1485	−0.4677	0.64
CellularityHigh	−0.0983	0.9064	0.1473	−0.6674	0.5045
ChemotherapyYES	−0.2141	0.8072	0.1563	−1.3703	0.1706
ER.StatusPositive	0.0338	1.0344	0.1482	0.2279	0.8197
Neoplasm.Histologic.Grade2	−0.0294	0.971	0.2026	−0.145	0.8847
Neoplasm.Histologic.Grade3	−0.2158	0.8059	0.2666	−0.8094	0.4183
HER2.StatusPositive	0.5025	1.6529	0.1377	3.649	3e−04
Hormone.TherapyYES	−0.2314	0.7934	0.1094	−2.115	0.0344
Inferred.Menopausal.StatePre	0.3634	1.4382	0.1588	2.2883	0.0221
Lymph.nodes.examined.positive	0.0479	1.0491	0.014	3.4247	6e−04
Mutation.Count	0.0057	1.0057	0.0093	0.6107	0.5414
Nottingham.prognostic.index	0.1858	1.2042	0.1035	1.7954	0.0726
PR.StatusPositive	0.0322	1.0327	0.1013	0.3178	0.7506
Radio.TherapyYES	−0.1954	0.8225	0.0941	−2.075	0.038
Tumor.Size	0.0109	1.011	0.0032	3.3662	8e−04
Tumor.Stage2	0.2585	1.2949	0.1271	2.033	0.0421
Tumor.Stage3	0.3734	1.4526	0.2338	1.5972	0.1102
age	0.0294	1.0298	0.0054	5.4886	0
Concordance	0.649	(se = 0.012)			
Likelihood ratio test	169.6	on 18 df	*p* = <2e−16		
Wald test	194.5	on 18 df	*p* = <2e−16		
Score (logrank) test	209.6	on 18 df	*p* = <2e−16		

(Carrasquinha, Veríssimo & Vinga (2018); Xue & Schifano (2017)) Table 5 indicates the values that we used in the creation of the RSF models for both datasets. In addition to these values in the table, log-rank splitting rule is implemented as default choice (Segal, 1988; LeBlanc & Crowley, 1993) and the type of bootstrap is selected as sampling without replacement. In contrast to Breiman’s random forests, sampling without replacement is the default choice in the function of rfsrc in the package randomForestSRC in R (Ishwaran & Kogalur, 2021). Therefore out-of-bag (OOB) means out-of-sample.

**Table 5:** Summarized values for the creation of RSF model.

**Hyperparameters**	**GBSG2**	**METABRIC**
Sample size:	480	888
Number of events:	216	534
Number of trees:	1,000	1,000
Forest terminal node size:	15	15
Average no. of terminal nodes:	23.143	46.013
No. of variables tried at each split:	3	4
Total no. of variables:	8	16
Resampling used to grow trees:	swor	swor
Resample size used to grow trees:	303	561
Analysis:	RSF	RSF
Family:	surv	surv
Splitting rule:	Logrank random	Logrank random
Number of random split points:	10	10
(OOB) CRPS:	0.1651	0.1676
(OOB) Requested performance error:	0.3200	0.3564

A Cforest model based on 1,000 tree was also fitted with the default settings in the function of cforest() in party package (Strobl et al., 2007; Hothorn et al., 2006; Strobl et al., 2008). After that, the survival probabilities for the hypothetic patients explained in ‘Datasets’ were predicted for the illustration of the results at the first sight.

Table 6 indicates the predicted survival for three hypothetic individuals at the quantiles of the time period of the datasets based on the proposed models. The quantiles (in days) for the datasets are 568, 1,084, 1,685 and 2,659 for GBSG2 data and 1,217, 3,020, 5,257 and 10,530 for METABRIC data. Both random forest approaches utilise 1,000 trees. Interestingly, compared to the models and hypothetic individuals the youngest one has the least chance to survival in time for the GBSG2 study. This may be due to the fact that this study has only 1 patient out of 686 patients at age 21. The mean age is 53.05 with the standard deviation of 10.12. Random survival forest model may fail if the study has insufficient information around the prediction. Except that the Cox PH model predicts a lower chance of survival throughout the study period. For the METABRIC data, the predicted survival probabilities based on ensemble learning approaches for the hypothetical patients are considerably close to the each other. The predicted survival probability plot can be seen in Supplemental Information Files for all time points and for both datasets.

**Table 6:** Survival predictions (in %) for hypothetic patients at the quantiles based upon the models.

		GBSG2			METABRIC		
**Model**	**Day**	**Age 24**	**Age 53**	**Age 73**	**Age 24**	**Age 53**	**Age 73**
**Coxph**	1. Quantile	0.77	0.77	0.81	0.93	0.89	0.80
	2. Quantile	0.58	0.59	0.64	0.86	0.77	0.61
	3. Quantile	0.42	0.43	0.50	0.76	0.62	0.41
	4. Quantile	0.21	0.22	0.28	0.32	0.14	0.03
**RSF**	1. Quantile	0.39	0.85	0.84	0.63	0.76	0.68
	2. Quantile	0.26	0.71	0.64	0.51	0.61	0.46
	3. Quantile	0.20	0.58	0.52	0.38	0.47	0.27
	4. Quantile	0.17	0.44	0.39	0.22	0.22	0.08
**Cforest**	1. Quantile	0.68	0.82	0.83	0.69	0.73	0.66
	2. Quantile	0.54	0.68	0.68	0.55	0.57	0.43
	3. Quantile	0.43	0.51	0.50	0.42	0.43	0.24
	4. Quantile	0.30	0.36	0.36	0	0	0

**Figure 1:**
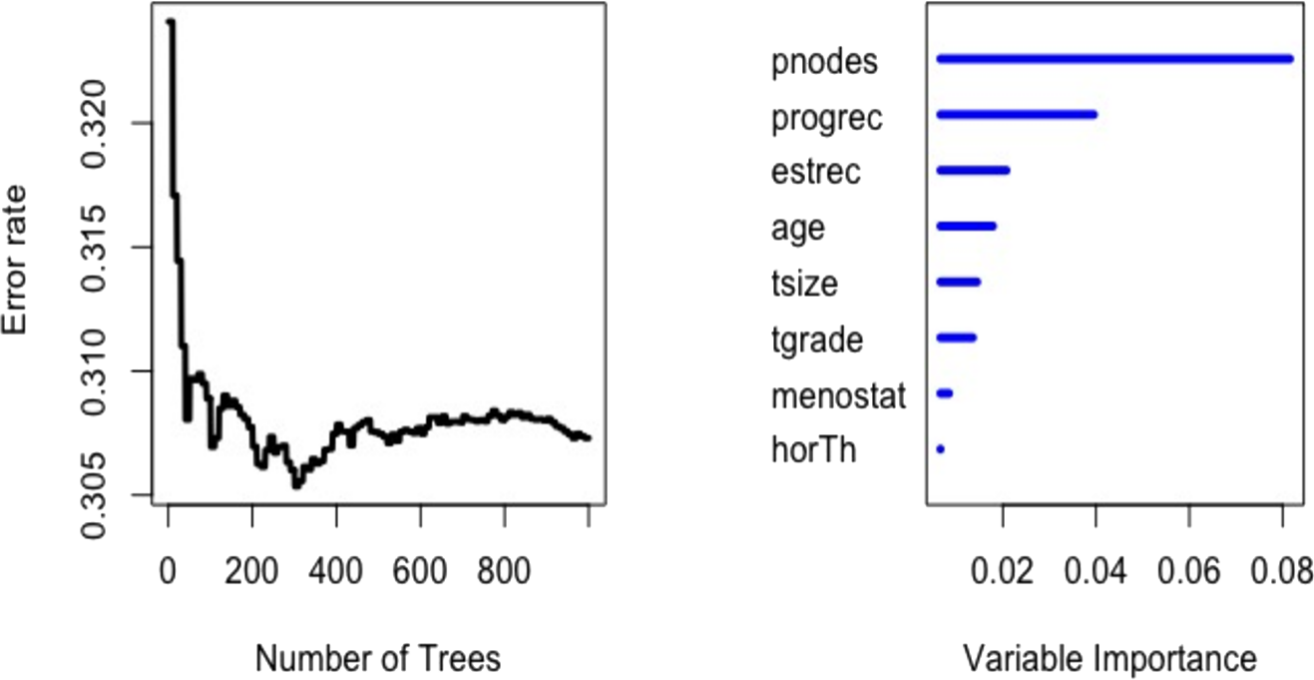
Error rate and out-of-bag importance values of the variables for log-rank splitting rule based on 1,000 trees in random survival forest for the GBSG2 data.

**Figure 2:**
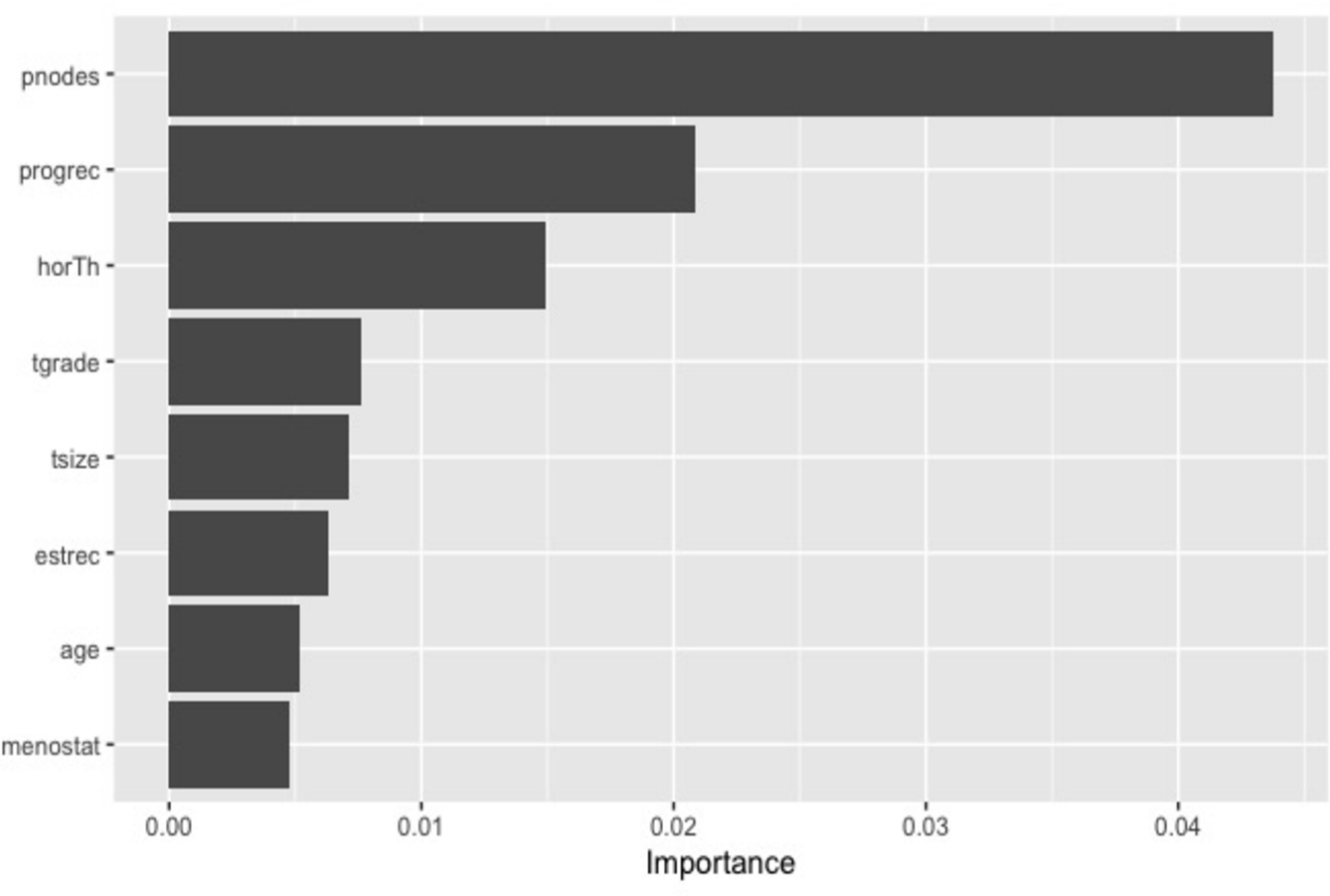
Out-of-bag importance values of the variables for log-rank splitting rule based on 1,000 trees in conditional inference forest for GBSG2 data.

The informativeness of each predictor for both of the random forest models is investigated for both datasets. Figure 1 (left panel) indicates the error rate for the RSF model as a function of the number of trees and the out-of-bag importance values for explanatory variables in GBSG2 study. Right panel presents the importance values for all the variables. The variable importance plot depicts that the six prognostic factors (*pnodes, progrec, estrec, tsize, tgrade* and *age*) had impact on recurrence free survival time. Nevertheless, substantially larger importance values had *pnodes* and *progrec* than the others. Figure 2 indicates the variable importance for the Cforest model. This model found the most significant variables as *pnodes, progrec* and *horTh*. *Pnodes* and *progrec* variables were found significant for all three models. Figure 2 is obtained using vip package (Greenwell & Boehmke, 2020).

**Figure 3:**
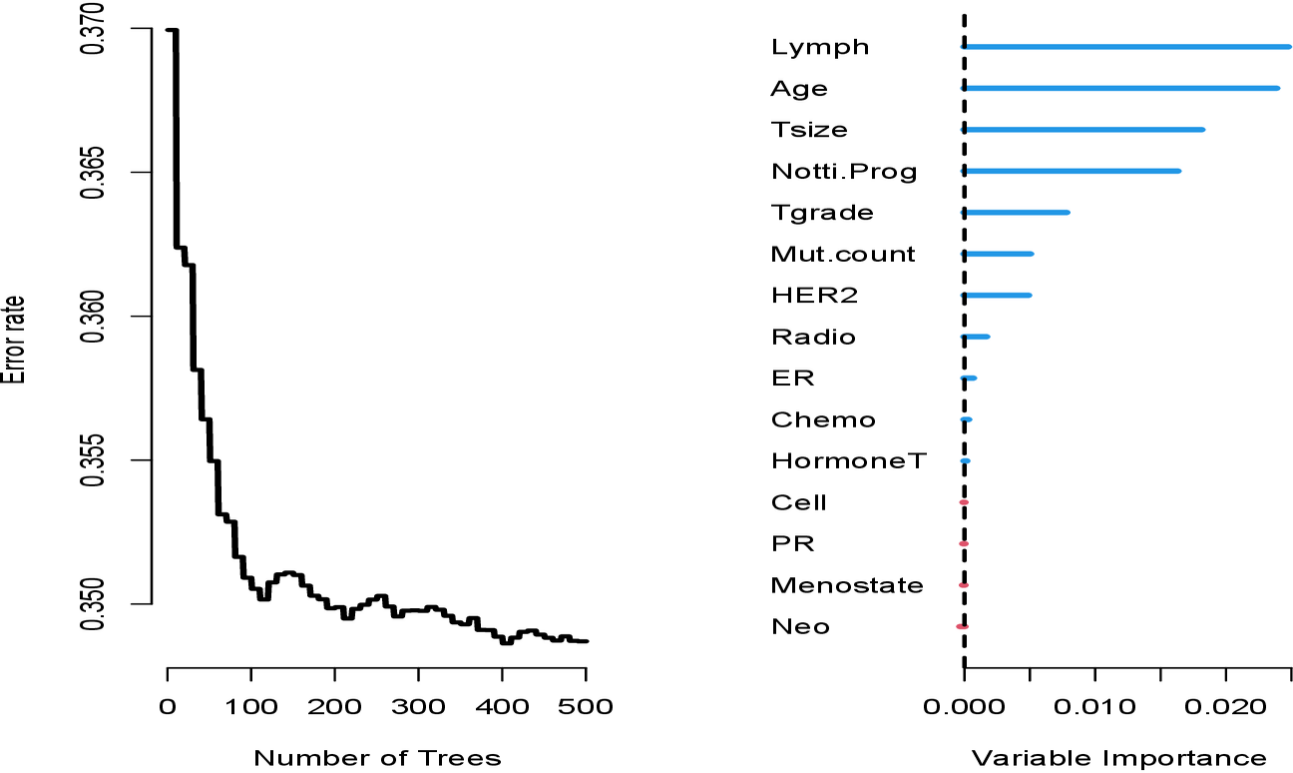
Error rate and out-of-bag importance values of the variables for log-rank splitting rule based on 1,000 trees in random survival forest for the METABRIC data.

**Figure 4:**
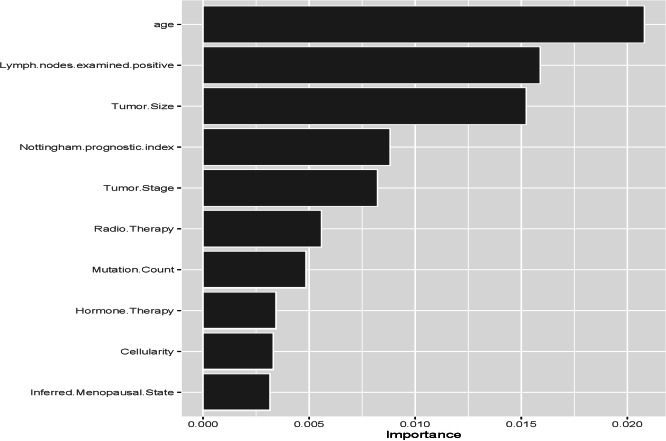
Out-of-bag importance values of the variables for log-rank splitting rule based on 1,000 trees in conditional inference forest for METABRIC data.

In order to the informativeness of the predictors for METABRIC data, Figs. 3 and 4 are created. Figure 3 (left panel) represents the error rate for the RSF model as a function of the number of trees and the out-of-bag importance values for features in METABRIC study. Right panel indicates the importance values for the variables. The variable importance plots for the ensemble learning models (Fig. 3-right panel and Fig. 4) depict that the five prognostic factors (*lymph node, age, tumor size, Nottingham prognostic index* and *tumor grade*) had effect on event free survival time with various order. Apart from Nottingham prognostic index the rest four variables are also statistically significant parameter of the Cox PH model (Table 4).

**Figure 5:**
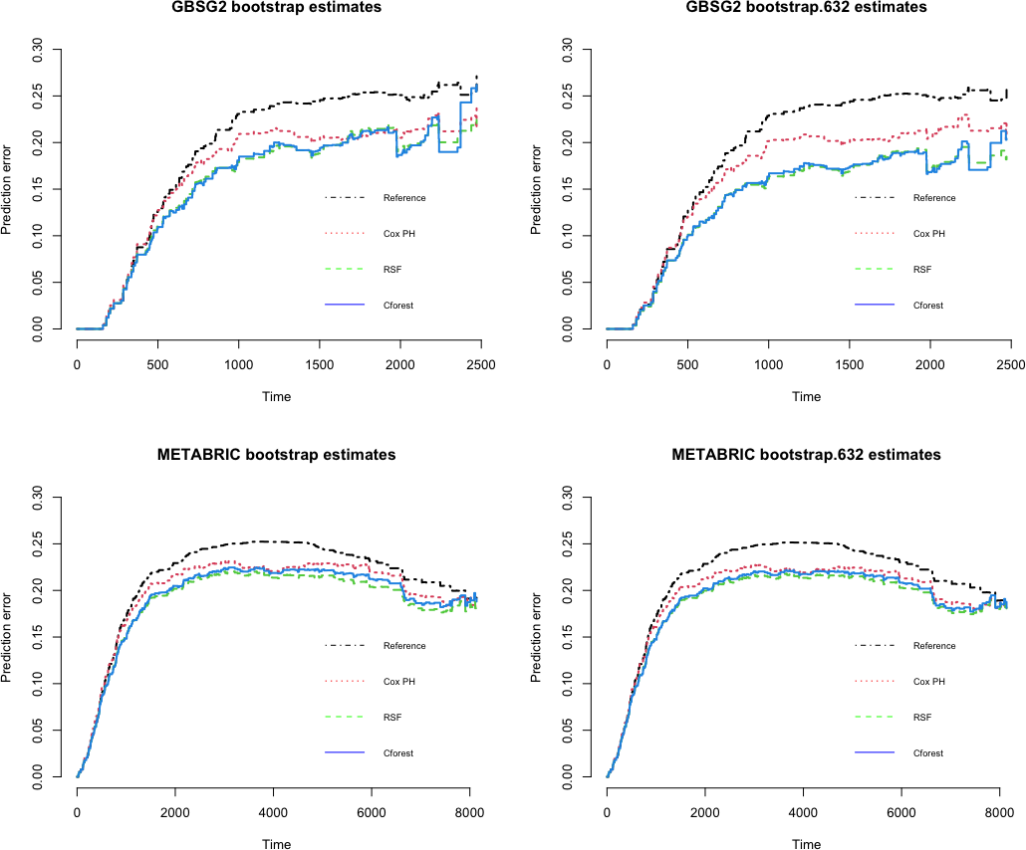
The prediction error curves based on the bootstrap cross validation (left panel) and the bootstrap .632 (right panel) estimations with 500 bootstrap samples for GBSG2 data (upper panels), for METABRIC data (lower panels).

Figure 5 depicts the integrated Brier score throughout the following time for the bootstrap cross-validation and the bootstrap .632 estimates of the prediction error based on 500 bootstrap samples. The error rates are computed based on the three modelling techniques and of the Kaplan–Meier as the reference model. The RSF and the Cforest methods are based on 1,000 tree per bootstrap samples and the size of each bootstrap samples is 200. The resampling is made without replacement. The prediction error rate of the three modelling approaches is very similar apart from the reference model for the bootstrap estimates (see the left panel in Fig. 5). The error rate of the reference model (the Kaplan Meier model) is higher than those three models, especially after day 1,000 for GBSG2 data, after day 2,000 for METABRIC data.

The RSF and the Cforest approaches are close to each other and they outperform the Cox PH and the reference model for the bootstrap .632 estimates for GBSG2 data (see the upper right panel in Fig. 5). However, the bootstrap .632 estimation method definitely improves models’ predictive ability. Apart from reference model, the performances of the rest models are quite close to each other in terms of METABRIC data (see the lower panels in Fig. 5). All four curves start at days 0 where all patients have no recurrence of the disease and all predictions are equal to 1.

Table 7 summarizes the integrated Brier scores in the ranges of time. Although the lowest score belongs to the RSF model for both the bootstrap cross validation and the bootstrap .632 estimates, both IBSs of random forest approaches are quite close to each other. Then the Cox PH model follows for both datasets. The performances of ensemble learning and the Cox models are considerably better than Kaplan–Meier model. The reference model has the highest error rate as seen in both Table 7 and Fig. 4. Nonetheless, the difference between the Cox PH model and random forest models of the bootstrap cross-validation estimates of the prediction error rate are smaller than the difference for the other method. This shows that the bootstrap sample size of 200 is not sufficient to indicate outperforming of the random forest approaches. Since the bootstrap .632 estimate method is a weighted linear combination of the bootstrap cross validation estimate and the apparent estimate, the wider difference between the forest approaches and the Cox PH model depends on the other strategy, the apparent estimate.

**Table 7:** IBS rates for both prediction estimation method for the last days available for all models based on 1,000 trees.

	GBSG2 data		METABRIC data	
**Model**	**IBS for bootstrap cross validation estimate**	**IBS for bootstrap .632 estimate**	**IBS for bootstrap cross validation estimate**	**IBS for bootstrap .632 estimate**
Reference	0.200	0.193	0.212	0.211
Cox PH	0.175	0.168	0.198	0.194
RSF	0.165	0.142	0.187	0.185
Cforest	0.165	0.143	0.191	0.188

**Figure 6:**
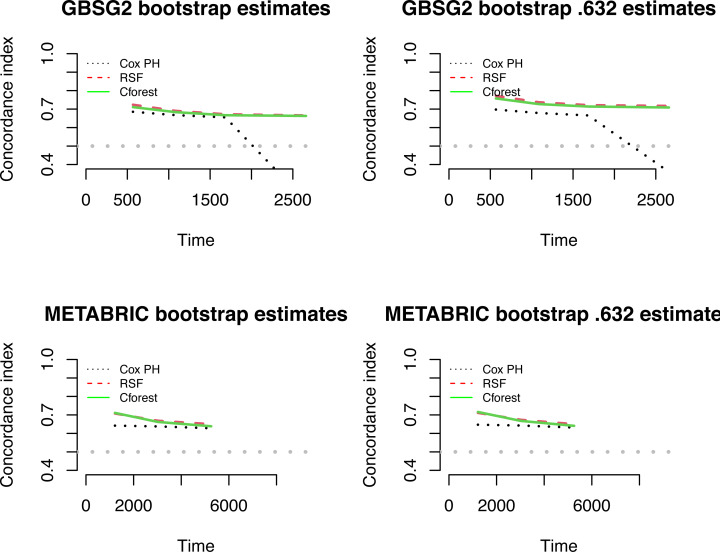
The concordance indexes for all three methods.

Figure 6 indicates the concordance index rates of the aforementioned three models for the bootstrap estimates (left panels) and the bootstrap .632 estimates (right panels) throughout the follow-up time for both datasets. The performances of the random forest approaches are almost the same, the Cox PH model has the lowest concordance rate for all the time, for both datasets and both estimation methods. The performances of the random forest approaches can be enhanced *via* the bootstrap .632 estimates as seen in the right panels in Fig. 6. Despite the very small difference in performances of these approaches, the RSF can be preferable due to its superiority. As bootstrap .632 estimation method is a weighted approach, this method varies from the bootstrap estimation method for first 1,000 days for METABRIC data. There is a sharp decrease of the C-index of the Cox model after around day 1,600 for GBSG2 data and day 5,000 for METABRIC data. This can be interpreted as the performance of Cox PH model cannot remain stable. However, this still needs further investigation and this is beyond the scope of this study.

## Discussion and Conclusion

In this study, we employed a Cox PH model and two different random forest-based approaches (RSF and Cforest) to predict the time-to-event in the population of breast cancer patients based on two datasets. We compared the performances of the models through the Harrell’s concordance index and prediction error curve with the two different bootstrap estimation methods. We also figured out the most important variables in the models as highlighted in ‘Results’.

The first objective of this study was to make evaluation of the performances of the aforementioned methods. The most obvious finding to emerge from the analysis is that both random forest approaches (RSF and Cforest) are superior to the Cox model for predicting the hazard of the event of the breast cancer patients based on both estimation methods. Nevertheless; the types of estimation methods can affect how well the methods perform. The bootstrap .632 estimation method clearly indicates the superiority of the ensemble learning based models in terms of concordance indexes (see Fig. 6) and prediction error curves (see Fig. 5). Both random forest approaches can detect complex, nonlinear structures amongst variables and this can be an advantage over the semi-parametric model. With these results the first objective is fulfilled. It can be concluded that the way of the estimation method can definitely improve the models’ predictive ability. So, the ensemble learning algorithms based on the bootstrap .632 estimation method have the higher performances in order to model the predicting risk of breast cancer survival.

These results agree with the findings of other breast cancer studies in literature (Table 8). Kurt Omurlu, Ture & Tokatli (2009) made a performance comparison between Cox PH model and RSF model. They analysed 279 patients diagnosed with breast cancer with various simulations. The authors concluded that RSF method yield better C-index values. They also figured out that tumor size and lymph nodes were the most important risk factors for breast cancer patients. In addition to this, the performance comparison of ensemble tree-based learner (RSF) and Cox PH model were done in some novel deep learning methods research, such as DeepHit method proposed by Lee et al. (2018), DeepSurv method proposed by Katzman et al. (2018) and NN-DeepSurv method proposed by Tong & Zhao (2022). They all used METABRIC dataset. Lee et al. (2018) concluded that the RSF method had the second highest C-index measure after DeepHit algorithm on METABRIC data. Katzman et al. (2018) concluded that their proposed method and RSF are capable of predicting individual’s risk and these methods outperform Cox PH model yielding higher C-index. However, Tong & Zhao (2022) stated that their proposed algorithm has the best performance with the highest C-index score among the other state-of-the-art methods for survival data. Moreover, Evangeline, Kirubha & Precious (2023) compared the DeepHit, RSF and Cox PH model performance on METABRIC dataset. They found that the DeepHit and RSF algorithms has superiority to Cox PH model with the higher C-index values. Nicolò et al. (2020) predicted metastatic relapse after surgical intervention with the Cox PH model and the RSF and concluded that the latter model had more accurate results with the higher C-index. However, most of them studies utilized only C-index as performance metrics, we used both C-index and PEC curves with both estimation methods. In addition to this, the predictive capability of the survival on breast cancer patients can be enhanced *via* the alternative estimation methods, such as bootstrap. When the C-indexes are compared with the existing studies on Table 8, the highest C-index value (∼0.74) belongs to this study apart from Evangeline, Kirubha & Precious (2023). In this study, the authors used an outcome variable (relapse-free status) as input variable. So, they found a significant correlation between the event status and this variable in nature, which is also explained subsequently in detail.

**Table 8:** Comparison with the existing studies.

**Author**	**Dataset**	**Model/ML algorithms**	**Best model**	**Evaluation metric of best model**
Kurt Omurlu, Ture & Tokatli (2009)	A dataset in Nicolò (2020)	Cox PH and RSF	Cox PH and RSF	C-index (∼0.29)
Lee et al. (2018)	METABRIC	Deephit, Cox PH, RSF, DeepSurv	Deephit	C-index (∼0.63)
Moncada-Torres et al. (2021)	Breast cancer data from Netherlands Cancer Registry (NCR)	Cox PH, RSF, SVM, XGB	XGB	C-index (∼0.73)
Katzman et al. (2018)	SUPPORT, WHAS, METABRIC, Rotterdam&GBSG	DeepSurv Cox PH, RSF	DeepSurv	C-index (∼0.65)
Ganggayah et al. (2019)	A breast cancer dataset from the University Malaya Medical Centre	Decision tree, SVM, RF, XGB	RF and XGB	Accuracy (∼82.7%)
Tong & Zhao (2022)	SUPPORT, WHAS, METABRIC, Rotterdam&GBSG	Cox PH, RSF, NN- Deepsurv	NN-DeepSurv	C-index (∼0.65)
Nicolò et al. (2020)	Metastatic relapse in early breast cancer data	Cox PH and RSF	RSF	C-index (∼0.66)
Evangeline, Kirubha & Precious (2023)	METABRIC	Cox PH, RSF, DeepHit	DeepHit and RSF	C-index (∼0.82)
Proposed work	METABRIC, GBSG2	Cox PH, RSF CForest	RSF and CForest	C-index (∼0.74)

The main contribution of this study was to demonstrate the ensemble learning algorithms to survival analysis performs better than the classical survival method (the Cox PH model) in predicting the risk of breast cancer patients and enhance the models’ predictive abilities and efficacy with the bootstrap and bootstrap .632 estimation method. All the results indicated that the estimation method has a significant impact on the algorithms’ performances.

The second objective of this study was to provide better understanding of the reasons performing the models the way they did. The variable importance plots (Figs. 1–4) show the difference between the ensemble learning models due to the importance levels among variables for both datasets. The first thing to note for GBSG2 study is that the most important two variables are the same for all three models (*pnodes* and *progrec*), while the third one varies. The third most important variable in Cox PH model can be either *horTh* or *tgrade*. It is *estrec* in the RSF model and *horTh* in the Cforest model. The unexpected finding is that the variable of *horTh* is the least important variable according to the results for the RSF model (see Fig. 1). In addition to this the Cforest model finds the variable of *estrec* is ranked at the bottom. The rest of the variables are somewhere in between. As for METABRIC study, all models indicate that lymph node, age and tumor size are the most important variables with the varying order. This means that older patients, with larger tumor size and more lymph nodes examined positive have increased risk of having the event. Those covariates affect the prognosis of the subjects. These results are consistent with the key factors specified in Roder et al. (2012) and Abbass et al. (2011). They stated that tumor size, positive nodal status and age are the among most important factors for prognosis of breast cancer. Evangeline, Kirubha & Precious (2023) stated that these variables have significant impact on the prognosis of the breast cancer; however, they found out that the most important variable with the 88% correlation is relapse-free status. We do not agree with this statement since the relapse-free status is an output so that this variable could be modelled as competing risk output instead of covariates. In order to maintain the consistency between datasets, we pooled the relapse-free status with the living status of patients as the new event output. In terms of informativeness of each predictor the models yield slightly different results thereby fulfilling the second objective is fulfilled.

The third objective of this study was to indicate the survival probability prediction results for the hypothetical patients. This objective is fulfilled *via* the illustration of the survival probability prediction results in the previous section. The most eye-catching point is that all methods perform similarly except for the youngest hypothetical individual in GBSG2 data. This may be due to the fact that there is very little information in the datasets for the young people. So that the Cox PH model has the smaller survival probability prediction than the other methods.

The comparison of the methods is made through two datasets, namely GBSG2 and METABRIC. In order to generalise these findings other settings may be needed with replications. Nevertheless, these results have important implications for enhancing the more complicated models including survival submodel such as joint models for longitudinal and survival data proposed by Wulfsohn & Tsiatis (1997), dynamic prediction of breast cancer through the random survival forest model (Buyrukoglu, 2024), super ensemble learning model (Doğru, Buyrukoğlu & Arı, 2023) and advanced joint models (Buyrukoglu, 2018). The main goal of this model is to detect the disease early and provide timely diagnosis, which are the two building blocks of the breast cancer management. The survival side of this model could be built up with an ensemble learning model and this will help other researchers in gaining a better understanding.

Prevention of cancer is one of the most critical health issues of this century, with the global burden. This research will gain insight into the development of the methodology to achieve the two pillars of this prevention: health promotion for early detection and timely diagnosis. The predictive and modelling capabilities of the models can enable researchers to utilize the ensemble learning models as a tool in the exploration and understanding of the disease etiology and prevention of the progress. These are the direct interest of the joint models for longitudinal and survival data, in which the survival side can be developed these two machine learning models.

## Supplemental Information

10.7717/peerj-cs.2147/supp-1 Supplemental Information 1 Comparison of RSF and cforest to Cox regression for GBSG2 data

10.7717/peerj-cs.2147/supp-2 Supplemental Information 2 Outlier detection of datasets

10.7717/peerj-cs.2147/supp-3 Supplemental Information 3 Comparison of RSF and cforest to Cox regression for METABRIC data

## Additional Information and Declarations

### Data Availability

The following information was supplied regarding data availability: The METABRIC data is available at https://www.cbioportal.org/datasets (Breast Cancer (METABRIC, Nature 2012 & Nat Commun 2016)). The GBSG2 data are available at GitHub and the R code are available at GitHub and Zenodo: – https://github.com/goncabuyrukoglu/Ensemble-Learning-models-for-breast-cancer-survival-prediction. – BUYRUKOGLU, G. (2024). Survival analysis in breast cancer: Evaluating ensemble learning techniques for prediction. In Peerj Computer Science. Zenodo. https://doi.org/10.5281/zenodo.11504093. The raw measurements are available in the Supplemental Files.
