# Supplementary material for: Survival analysis in breast cancer: evaluating ensemble learning techniques for prediction
Source: PeerJ Comput Sci. 2024 Jul 10;10:e2147. doi: 10.7717/peerj-cs.2147 (PMC11323082; doi:10.7717/peerj-cs.2147)
Supplement: Supplemental Information 2 [file peerj-cs-10-2147-s002.docx]

**S 1. Outlier Detection**

**Figure 1** Martingale residuals against linear predictors for both datasets

In terms of outlier detection in the datasets, a plot of Martingale residuals against linear prediction is very practical [1]. It helps to identify the subjects who have event very soon or do not have event [2]. Figure 1 indicates the Martingale residuals against linear predictor for both datasets, in order to examine the potential outliers. In GBSG2 study, there are 3 outliers with the lowest Martingale residuals as shown in the left panel in Figure 1: Patient 116, 163, 42. They are scrutinized carefully. They do not have the event and do not receive the hormonal therapy. The reason why they are detected outlier is that the number of positive nodes is the most important variable in the model as can be seen in Table 3, variable importance figures subsequently. In METABRIC study, there are two outliers: Patients 278 and 830. Patient 278 is 61 years old with tumor size 50mm and number of 15 positive nodes. Patient 830 is 63 years old with tumor size 22mm and mutation count 24. This patient lived unexpectedly long. Despite the fact that these outliers may affect the models’ performances, excluding them from the analysis would be unrealistic. This is commonly encountered problem in the real-life problems, so that we keep them as they are.

**S 2**

In order to illustrate a construction of the three hypothetical new individuals is made. These hypothetic patients have the same variables as in the GBSG2 study with different ages: They do not receive hormonal therapy, have 3 positive nodes, their tumor size is 25 mm, their tumor is second grade, their progesterone receptor is 32 fmol, estrogen receptor is 36 fmol. Their ages are 24, 53 and 73 and the younger one is on premenopausal status, the rest are on postmenopausal status.


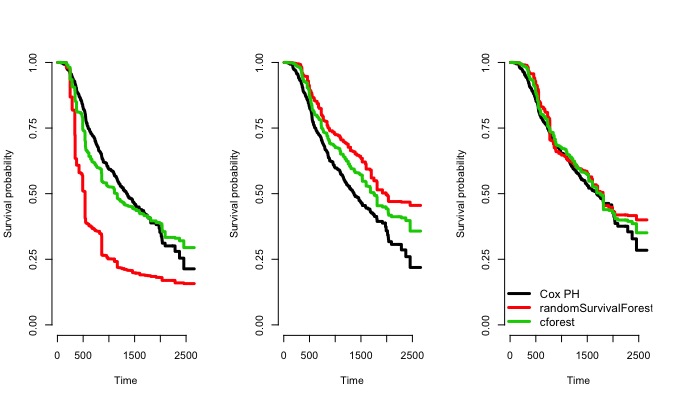


**Figure 2** Survival curve predictions based on GSSG2 dataset for hypothetic Aged 24 patient left panel, Aged 53 patient middle panel, and Aged 73 patient right panel

A new data construction has been made for the illustration purpose of the METABRIC data. Three hypothetical patients have been created with the same characteristics apart from age variable.

**Figure 3** Survival curve predictions based on GSSG2 dataset for hypothetic Aged 24 patient left panel, Aged 53 patient middle panel, and Aged 73 patient right panel

Figure 2 and Figure 3 present the survival curves prediction for the patients for a given modelling strategy. The prediction is made through the all event times. The models yield nearly the same results for the old patients. The survival predictions of the RSF and Cforest models for the middle age do not differ much in GBSG2 study. For this patient the Cox PH model is more extreme. For the METABRIC study, the survival predictions of the patients based on the ensemble learning models nearly the same, whereas the Cox PH model has the highest survival probability predictions for all the subjects.

[1] E. Carrasquinha, A. Veríssimo, and S. Vinga, “Consensus outlier detection in survival analysis using the rank product test Survival Analysis Cox regression model,” PLoS One, 2018, doi: 10.1101/421917.

[2] Y. Xue and E. D. Schifano, “Diagnostics for the Cox model,” Commun Stat Appl Methods, vol. 24, no. 6, 2017, doi: 10.29220/CSAM.2017.24.6.583.
